# Flower-like Superhydrophobic Surfaces Fabricated on Stainless Steel as a Barrier against Corrosion in Simulated Acid Rain

**DOI:** 10.3390/ma15207104

**Published:** 2022-10-13

**Authors:** Regina Fuchs-Godec

**Affiliations:** Faculty of Chemistry and Chemical Engineering, University of Maribor, 2000 Maribor, Slovenia; regina.fuchs@um.si

**Keywords:** acid rain, corrosion, flower-like structure, inhibition, stainless steel

## Abstract

Functionalisation of the metal surface of low-carbon ferritic stainless steel (from hydrophilic to hydrophobic properties) was achieved by flower-like hierarchical structures on a steel substrate prepared by a low-cost immersion method. The flower-like structured hydrophobic layers on the steel substrate were obtained by immersing the samples in an ethanolic solution of stearic acid with the addition of various concentrations of expired vitamin E ((+)α-tocopherol). The stability and corrosion-inhibiting effect of the hierarchically structured (such as natural cornflower) hydrophobic layers were studied systematically during short and long immersion tests, 120 h (five days) in an acidic environment (pH = 3) using potentiodynamic measurements, electrochemical impedance spectroscopy and chronopotentiometry. The surfaces of the samples, their wettability, surface morphology and chemical composition were characterised by contact angle measurements, SEM, ATR-FTIR and EDAX. After 120 h of immersion, the inhibition efficiency of the flower-like structured hydrophobic layers on the steel substrate in the selected corrosion medium remained above 99%, and the hierarchical structure (flower-like structure) was also retained on the surface.

## 1. Introduction

Corrosion is a global problem that we still cannot comprehend despite all the knowledge, progress and technology [1]. The only thing we can be proud of is that we are already able to mitigate the destructive effects of corrosion processes largely, reduce the rate of corrosion significantly, and, thus, extend the life of building materials and materials for everyday use.

The trend is towards the creation of new materials or the protection of existing materials, which are indispensable in industry in various sectors. Most of the protection is in the direction of protective coatings, paints, smart coatings, self-repairing coatings, protective films formed by adsorption processes, functional surfaces (different types of hydrophobic and superhydrophobic surfaces), etc. [2,3,4,5,6,7,8,9,10,11,12,13,14]. The “green orientation” and the increasingly strict European Directives to use only non-hazardous substances for the formation of protective coatings have given further impetus to research in this field. In addition, the trend is increasingly towards a circular economy or zero or zero-waste operations. In other words: What is no longer usable in a certain area (due to an expiration date-food supplements) does not have to be thrown away, but can be a valuable raw material that can be used for other purposes, i.e., “every waste can be a raw material”. In the field of green protective coatings or green corrosion protection, much research has been performed on the incorporation of vitamins as potential green inhibitors in quite aggressive media on various metallic materials [15,16,17,18,19,20,21,22,23,24,25,26,27,28]. Among the water-soluble vitamins, there are studies showing the great potential of vitamin C and a wide range of B vitamins as corrosion inhibitors for various metallic materials. The inhibition efficiencies achieved are over 90% even in highly aggressive acidic media [16,17,21,25].

The great potential of green corrosion inhibitors can be attributed to the group of fat-soluble vitamins, which includes vitamin E ((+)-α-tocopherol, E307). Vitamin E is fat soluble, which characterises it as a non-polar substance, and gives it the ability to participate in the formation of hydrophobic layers or superhydrophobic surfaces that can provide excellent corrosion protection [26,27,28]. Superhydrophobicity is an effect in which the surface roughness and chemical composition combine to form an unusually hydrophobic surface that repels water/electrolyte and forms a successful barrier that protects the surface from electrolyte penetration and corrosion reactions. Superhydrophobic surfaces have been studied and formed successfully on various metallic surfaces (stainless steel, Mg, Cu, Al, and Zn) in recent years [29,30,31,32,33,34,35]. There are a number of methods for producing superhydrophobic surfaces, such as the sol–gel method [36], arc-sprayed coating [37], laser texturing [38,39], and the hydrothermal method [40,41], with a combination of plasma surface and heat treatment, or as base-coats and top-coats [42]. However, the trend shows that fast, simple, and economical methods for achieving the goals are always in the foreground. 

The next interesting feature of the superhydrophobic layers that interests both the scientific and academic communities is their hierarchical structure. The SEM images showed that these hierarchically structured superhydrophobic layers resemble flowers (a flower-like structure). The lotus leaf and the so-called lotus effect are natural examples of superhydrophobicity. The superhydrophobicity of the lotus leaf is due mainly to the low surface energy provided by the epicuticular wax crystalloids and the air pockets trapped in the micrometre-sized papillae structure, which minimises the contact area between the solid and the water [43]. It is now known that superhydrophobicity can be achieved by combining high surface roughness with low surface energy coating. The methods for producing a superhydrophobic surface can be divided into two main approaches: In the first step, a rough structure is created on the substrate surface (by etching, laser, plasma etching, etc.), and, in the second step, the rough surface is modified with a special low surface energy material such as fluorochemicals and silicones (silane, siloxane, fatty acids, natural wax) [12,14]. In order to achieve the highest possible hydrophobicity, and, at the same time, the highest possible adhesion, the way the surface is structured after etching, laser treatment, etc., is of great importance. The distances between the “peaks and valleys” of the needle-shaped structure thus created must not be too large (micro and nanometre scale structures), because only these and the subsequent coating/layers with a special material with low surface energy prevent the penetration of the water solutions (corrosive medium), so that the base material (metal) remains undamaged in the case of corrosion processes. Only air can be trapped in the resulting voids/pockets of the needle-like structure, further creating a barrier between the metal surface and the medium present [44,45,46,47,48,49]. 

In relation to everything mentioned thus far, the idea of our research was to combine the ideas of the circular economy (waste is a raw material), green inhibitors, superhydrophobic surfaces, with the challenge of creating hierarchical structures in the form of flowers due to their exceptional properties (corrosion protection properties of interest to us), and the use of inexpensive and simple methods to obtain corrosion resistant coatings/coatings on various metal materials in the media most commonly involved in corrosion research (simulation of seawater 3.0–3.5 wt% NaCl, simulated form of acid rain) and their durability over the longest possible period. The selected system was a metallic material (Cu, Cu40Zn, low carbon stainless steel AISI 410S) and the corrosion medium was a simulated form of acid rain acidified to pH 5 in the case of Cu, Cu40Zn, and to pH 3 in the case of stainless steel (SS). For fabrication of superhydrophobic self-assembled protective layers, we chose an ethanolic solution of 0.05 M (mol L^−1^) stearic acid (SA) and a combination of stearic acid at the same concentration, and fat-soluble vitamin E (E307) with an expired date in three different concentrations (0.5 wt%, 1.0 wt%, and 2.0 wt%), and a simple immersion method. Why stearic acid and why a concentration of 0.05 M? It is known that stainless steel contains at least 10.5% chromium. This chromium reacts rapidly with the surrounding oxygen and forms a thin oxide layer on the surface of the steel, and, likewise, copper. The SA molecule consists of a carboxyl head group and a long hydrophobic alkyl chain. It has been reported that the carboxyl group can react with metal oxides and form a chelate bond with the metal atom. Therefore, the absorption of SA on the surface of the Cr_2_O_3_ or CuO layer is strong [50,51]. Tao Liu et al. have reported that the flower-like surface structure is formed on the Cu surface in the solution SA with a concentration of 0.06 M. At a lower concentration, flower-like clusters cannot be formed, and at a higher concentration, a non-resistant structure is formed, which can easily be rinsed off with water [51]. 

In our studies, we used an SA concentration of 0.05 M and the flower-like structure was obtained, while, at a concentration of 0.01 M SA, we did not obtain this type of structure, which confirms their results. In the field of corrosion research, where inhibitors of one kind or another are used (added to the solution or to the protective layer), it is important to know that, in some systems, an inhibitor really acts as an inhibitor, while in others it may be an activator, depending on the material chosen and the corrosive medium with which it may react. Various computational studies using computer modelling (the Monte Carlo model) can be of great help, as they can help to explore very complex structures, properties and processes at the atomic level that are difficult or impossible to access using experimental techniques. They can also narrow the range of materials that can be bound to the surface easily and efficiently, saving a tremendous amount of material and time in performing experiments [52,53,54]. However, in the final phase, experiments in a physical form are still very important and crucial to confirm the applicability of the introduced process and the resulting protection on a larger scale.

**Figure 1:**
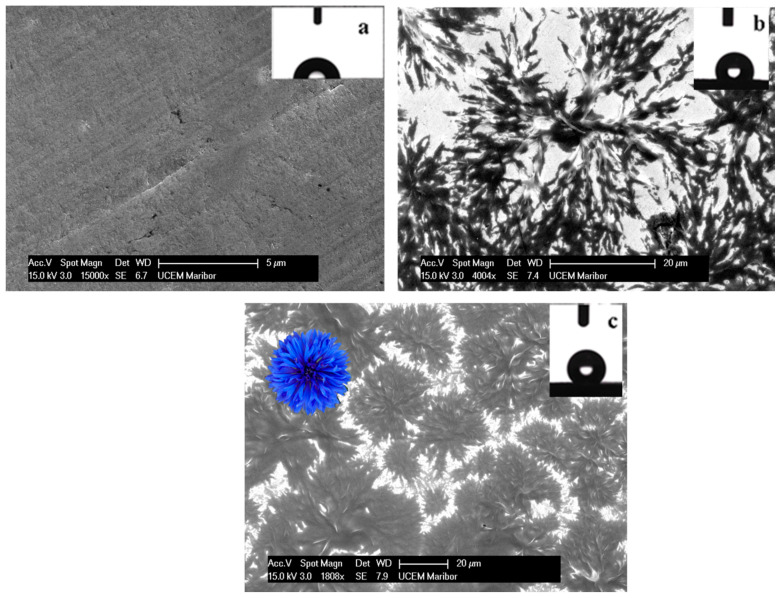
SEM of the untreated and modified surface of SS type AISI 410S. (**a**) Untreated and etched surface (CA = 71.5° ± 3). (**b**) Modified surface in an SA solution for one hour (CA = 122.8° ± 2). (**c**) Modified surface in an SA solution with the addition of 2.0 wt% of E307 for one hour (CA = 138.7° ± 3) [27].

The studies presented here extend the results already obtained in this field, to summarise briefly. In our first study in this field, such layers were formed successfully on copper and Cu40Zn (bronze). The formed hydrophobic layers with a contact angle value close to 150° (which corresponds to the value for superhydrophobicity), consisted of only 0.05 M SA, and, in combination, 0.05 M SA with the additive of E307 in concentrations of 0.5, 1.0 and 2.0 wt%, as mentioned above. The SEM images showed a hierarchical structure with a not too pronounced flower-like structure. The results of the inhibition efficiency in corrosion media of simulated urban rain (pH = 5) were surprising, and they exceeded 99% in the case of the addition of E307 [26]. Such a result definitely forces us to proceed with other systems, and thus, we extended the research to stainless steel (SS) of the AISI 410S type in a 3.0 wt% NaCl solution after short-term exposure and a long-term test. Under the same conditions of fabrication of superhydrophobic protective layers as described previously, layers with a hierarchical structure in the form of a true flower structure were formed on the surface of the SS AISI 410 s, as shown in Figure 1. Moreover, a high inhibition efficiency of >99% was achieved again for the selected system when the as-prepared self-assembled superhydrophpobic layers were formed in an ethanol solution of SA with the addition of E307 [27,28]. 

Since this type of material is classified as a construction material, we decided to extend this study to the corrosive environment of acid rain with a pH of 3. This is the acidity level at which this type of stainless steel starts to lose its corrosion resistance at the expense of the formed passive film, and the protection of the material is definitely necessary. This is the main reason and goal to why we thought that it would be useful to carry out the study in such a corrosion medium and investigate how successful the protection by such coatings/layers could be, or rather how the durability of this type of layer and the stability of the flower-like structure itself was during shorter and longer exposure in the selected corrosion medium. Three different electrochemical methods were used for the experimental results, namely, classical potentiodynamic measurements, chronopotentiometric measurements and electrochemical impedance spectroscopy (EIS), while the surfaces of the samples, their wettability, surface morphology and chemical composition were characterised by contact angle measurements, SEM, ATR-FTIR and EDAX.

## 2. Materials and Methods

### 2.1. Materials and Chemical

The low carbon ferritic stainless steel (AISI 410S, Zelezarna Ravne, Slovenia) used in the experiments contained 0.04% C, 0.471% Si, 0.02% S, 13.2% Cr, 0.307% Ni, 0.213% Cu, 0.416% Mn, 0.007% Al, 0.141Mo, and 0.024% P, the balance being Fe. The following chemicals were used to prepare the test solution, i.e., the simulated acid rain, NaNO_3_ (Merck GesmbH, Graz, Austria, CAS number 7631-99-4), NaHCO_3_, (J.T. Baker, Phillipsburg, NJ, USA, CAS 144-55-8) and Na_2_SO_4_ (Merck GesmbH, Graz, Austria, CAS number 7757-82-6). The solution itself was acidified to pH 3 with H_2_SO_4_. The stearic acid used (Sigma-Aldrich, Darmstadt, Germany CAS number 57-11-4, St. Louis, MO, USA) was of pure grade (95%), while the (+)-α-tocopherol (vitamin E, E307) was also a pure product (97%) from Sigma-Aldrich, Darmstadt, Germany (CAS number 59-02-9). All the chemicals were used as received and without further processing.

### 2.2. Devices

The present investigation includes measurements on the following apparatus:

- Electrochemical measurements:
- Gamry 600™potentiostat/galvanostat, Warminster, PA, USA controlled by an electrochemical program.
- Processing and analysis of experimental data using the following software programs: CorrView, CorrWare, Zplot and ZView programs from Scribner Associates, Southern Pines, NC, USA (all version 2.80).
- ATR-FTIR: SHIMADZU-IRAffinity-1, Shimadzu Europa GmbH, Duisburg, F.R. Germany.
- Scanning Electron Microscope (SEM): FEI Sirion 400 NC, Eindhoven, the Netherlands.
- Goniometer: Data Physics OCA 35, Filderstadt, Germany.

### 2.3. Preparation of the As-Prepared Hydrophobic Layers

**Scheme 1:**
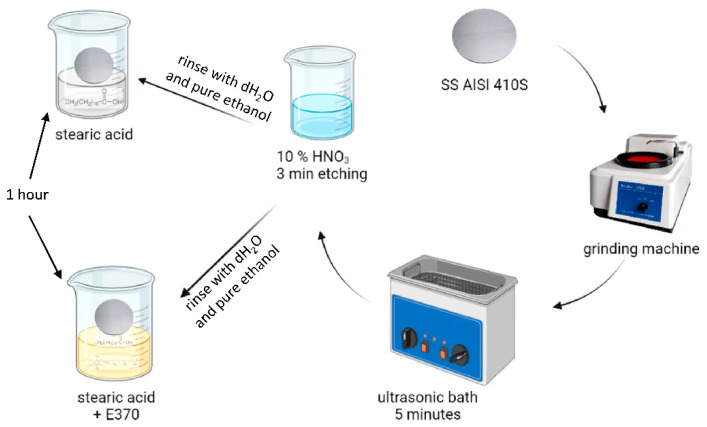
Schematic diagram of sample preparation and fabrication of the as-prepared self-assembled superhydrophobic layers.

The AISI 410S stainless steel specimens (⌀ = 1.2 cm) were first ground with various grits of emery paper (320, 400, 600, 800, 1000, and 1200), cleaned ultrasonically with alcohol and deionised water and dried with compressed air. To create a superhydrophobic surface, we used a simple immersion method. The samples were etched chemically in 10% HNO_3_ for 3 min, after which the etched samples were rinsed quickly with deionised water followed by pure ethanol, and immersed immediately in an ethanol solution of 0.05 M SA or in a mixture of 0.05 M SA + x wt% E307, x = (0.5, 1.0, and 2.0) wt% (hereafter wt% = %) at room temperature for 1 h. The modified surfaces of SS thus obtained were dried with compressed air to perform further characterisation or electrochemical measurements. The immersion time was constant, one hour, as was the concentration of the stearic acid, c = 0.05 M. The entire sample preparation procedure is shown in Scheme 1.

### 2.4. Electrochemical Characterisation

The electrochemical measurements were carried out by chronopotentiometric measurements, i.e., monitoring of the open circuit potential (OCP) in different time windows of 1, 10, 25 and 120 h (5 days). Furthermore, the potentiodynamic polarisation measurements and electrochemical impedance measurements (EIS) were employed usefully to evaluate the behaviour of the as-prepared self-assembled hydrophobic layer, and to calculate the inhibition effect based on characteristic corrosion parameters. A simulated form of acid rain with the composition 0.2 g/L Na_2_SO_4_, 0.2 g/L NaHCO_3_, and 0.2 g/L NaNO_3_, acidified to pH = 3 with H_2_SO_4_ was used as the corrosive medium. The electrochemical measurements were performed with a three-electrode arrangement. A saturated calomel electrode (SCE) was used as the reference electrode. A platinum electrode served as the counter electrode. The treated and untreated metal samples with an exposed area of 0.875 cm^2^ were used as the working electrode. Electrochemical impedance spectroscopy measurements were performed in a frequency range from 10^6^ to 10^−3^ Hz at a constant open circuit potential (OCP) perturbed by an amplitude of 10 mV (peak to peak). All the experiments were performed at 25 °C ± 1. Similar to the OCP measurements, the EIS measurements were performed after 1, 10, 25, and 120 h of immersion in a simulated solution of acid rain, while the potentiodynamic measurements were performed after one hour of immersion and 120 h in the selected corrosive medium.

### 2.5. Surface Characterisation

#### 2.5.1. Contact Angle

The CA measurements were performed using a contact angle system from Data Physics OCA 35 (Filderstadt, Germany)) in a thermostatically controlled room at 25 °C ± 1. A drop of ultrapure water (3 μL) was dropped on the modified and unmodified stainless steel surfaces (AISI 410S). The average value CA was determined by measuring five replicates for each sample.

#### 2.5.2. ATR-FTIR Analysis

The ATR-FTIR method was used to test whether the composition of the self-assembled hydrophobic layer changed due to prolonged exposure to a corrosive environment. The recorded IR spectra were identified by comparison with the standard positions of the group peaks. The spectra for the hydrophobic layer (SA + 2.0% E307) were compared before and after 120 h of immersion in a simulated acid rain solution. The IR spectra were recorded with a spectral resolution of 4 cm^−1^ and a wavenumber in the range 400–4000 cm^−1^.

#### 2.5.3. Surface Investigation by SEM/EDX

The scanning electron microscope (SEM) (XL FEG/SFEG/SIRION) was used to characterise the morphology of the surfaces of the self-assembled layers. SEM was coupled with an EDAX unit. For all the investigations, the beam energy was 15 and 20 kV to achieve excitation of all elements.

## 3. Results and Discussion

### 3.1. Morphology of the As-Prepared Hydrophobic Layers

#### Contact Angle Measurements

**Figure 2:**
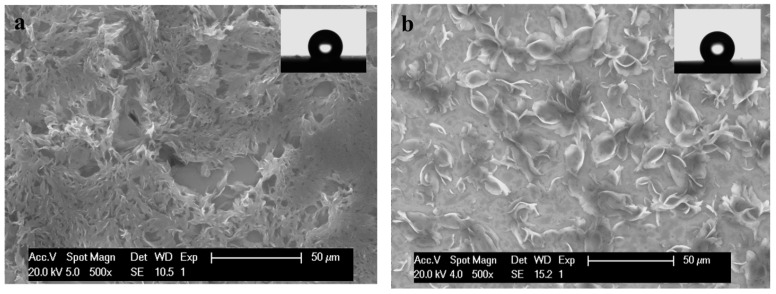
SEM-modified surface of SS type AISI 410S in an SA solution. (**a**) Without (CA = 117.8° ± 2). (**b**) With the addition of 2.0% of E307 after 120 h testing in a simulated solution of acid rain with pH = 3 at 25 °C (CA = 137.2° ± 1).

The contact angle of a liquid droplet is a suitable means of describing the wetting properties of a surface. In our previous research, it has already been shown that hydrophobic layers formed in this way can be classified as highly hydrophobic layers, with contact angle values close to 150° (so-called superhydrophobic layers) [26,27,28]. The CA values after prolonged exposure and whether the basic structure of these layers changed after 120 h of immersion in corrosion medium (simulated acid rain) were the main objective of the contact angle measurements and taking SEM photos. In our previous work, the change in morphology of these coatings on the same material was observed after prolonged exposure to 3% NaCl [27]. Therefore, the surface wettability of the coatings was determined by measuring the contact angle of the water (CA), spreading 5–8 drops over the entire surface to obtain reliable CA values. Figure 1 shows the surface morphology and wettability (the small squares indicate these drops) of the as-prepared self-assembled hydrophobic layers, with and without the addition of E307 in an ethanol solution of SA. The SEM images in Figure 1a–c show these hydrophobic layers before the electrochemical measurements, i.e., immediately after their formation, while in Figure 2a,b, the treated samples were exposed to a corrosive medium (acid rain, pH = 3) for 120 h. More specifically, Figure 1 shows SEM images of the morphology of (a) the untreated SS substrate, while Figures (b) and (c) show the modified SS surfaces exposed for 1 h in the SA solution and 1 h in the SA solution with the addition of 2.0% E307. We can emphasise that the morphology of the SS sample shown in Figure 1c clearly shows the hierarchical structure of the as-prepared self-assembled hydrophobic layer with a flower-like structure resembling the shape of a natural cornflower (blue flower). In addition, the SEM image (Figure 2a) shows the surface morphology of the as-prepared hydrophobic layer formed in an ethanol solution of SA after 120 h of immersion in the selected corrosive medium, and Figure 2b shows this layer formed in (SA + 2.0% E307), also after 120 h of exposure to a simulated acid rain solution (pH = 3). For both structures, after 120 h of immersion, it can be said that the hierarchical surface structure was still present, although the sharpness of the reliefs was weakened. The so-called flower-like structure was still present in the case (SA + 2.0% E307), with more rounded lines, which could be due to water-uptake, possibly also to swelling when the pores (micropores) were filled with the external electrolyte. The high degree of inhibition and the high contact angle values, even after 120 h of immersion, can be attributed to the fact that the hierarchical structure of the as-prepared hydrophobic layer was maintained, or only slightly changed, with respect to the initial state. As already mentioned, hierarchical structuring of the surface is usually a prerequisite for the occurrence of superhydrophobicity [55].

### 3.2. Electrochemical and Corrosion-Resistance Measurements

Classical potentiodynamic measurements and electrochemical impedance spectroscopy (EIS) were performed to evaluate the corrosion resistance of the metal matrix SS AISI 410S and the further modified stainless steel surfaces in a simulated acid rain solution at pH 3. A chronopotentiometric method was also included for measuring the open circuit potential (OCP). Like the EIS method, this method is considered non-destructive, because the OCP is a potential at which no current flows [56]. It does not provide information about the kinetics at the surface, but its value and trend over time are important. A negative trend in OCP with time (towards more negative values) indicates a tendency for the metal/surface to corrode in the solution, while a less negative OCP (reverse trend) indicates a low tendency for corrosion, indicates the formation of a protective layer (passive layer), or demonstrates the stability of the previously formed protective layer.

#### 3.2.1. Open Circuit Potential Measurements

**Figure 3:**
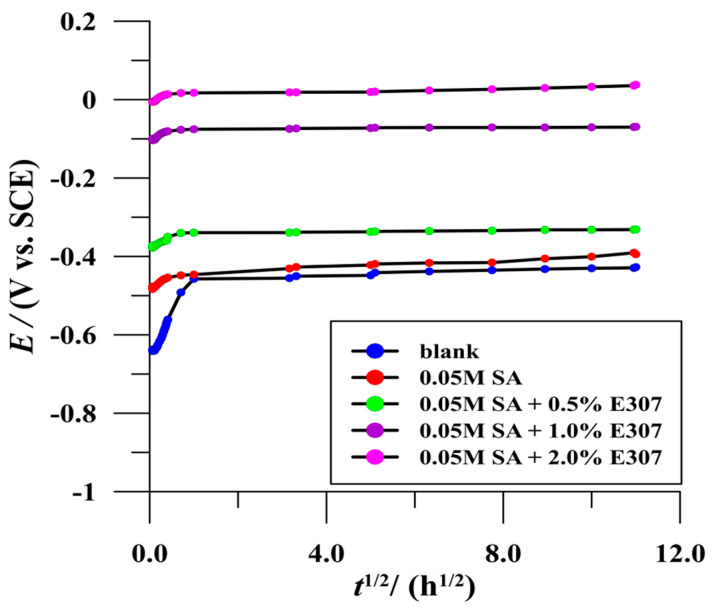
OCP vs. time curves (t ½/h½) for SS type AISI 410S for bare and modified surfaces in a simulated solution of acid rain with pH = 3 at 25 °C after 1, 10, 25, and 120 h of immersion.

The open circuit potential (OCP) was determined for the treated and untreated surfaces of SS AISI 410S in a simulated solution of acid rain with a pH of 3. The change in OCP was monitored over a period of 120 h. In all cases, an increasing tendency of the OCP towards a less negative value was observed, even in the case of the untreated surface (Figure 3). In the case of the untreated surface, the OCP value stabilised after one hour of exposure to the selected corrosive medium, indicating the beginning of the formation of the protective passive layer. A compact passive layer was formed only after prolonged exposure after 25 h, which was confirmed by the impedance spectroscopic measurements (Section 3.2.3). In the case of the modified SS surfaces (the self-assembled hydrophobic layer of SA with and without the addition of E307), stabilisation occurred much faster, between 240 and 600 s. In the cases presented, it was fastest when the surface was modified with (SA + 2.0% E307) and slowest with SA alone. Moreover, a stronger shift of the OCP values was observed towards less negative values. Such OCP response already predicts good inhibitory properties of the thus as-prepared self-assembled hydrophobic layer, even in such acidic medium pH = 3. According to the OCP value, the layer consisting of (SA + 2.0% E307) could be considered the highest quality of all, and the layer consisting of (SA + 1.0% E307) can be rated equally high. The OCP values for these two layers were different from the values for the other three layers. The OCP values of the passive layer and the hydrophobic layer (SA only) did not differ significantly, perhaps in stabilisation time, but the stabilisation of the OCP was faster in the case of SA. However, the good influence of E307 on the quality of the hydrophobic layer can already be observed in the case of the hydrophobic layer (SA + 0.5% E307). The value of the OCP was somewhere between the other two groups. Considering the OCP value, it can be assumed that the added concentration of E307 was too low for the selected corrosive medium to achieve sufficient protection.

#### 3.2.2. Potentiodynamic Polarisation Test

**Figure 4:**
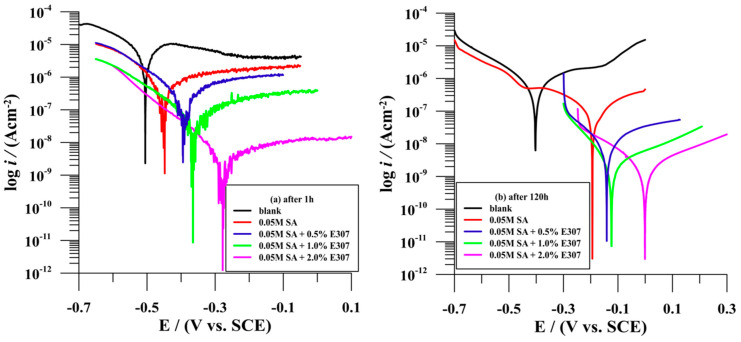
Potentiodynamic polarisation curves (1 mVs^−1^) for SS type AISI 410S for bare and modified surfaces in a simulated solution of acid rain with pH = 3 at 25 °C. (**a**) After 1 h of immersion. (**b**) After 120 h of testing. (The modified surfaces were prepared by immersion of SS type AISI 410S in 0.05 M stearic acid in ethanol, with and without the addition of E307).

Classical potentiodynamic measurements and electrochemical impedance spectroscopy (EIS) were performed to evaluate the corrosion resistance of the metal matrix SS AISI 410S and the further modified stainless steel surfaces in a simulated acid rain solution at pH 3. The polarisation curves of the unprotected surface of SS AISI 410S and the surface modified with self-assembled hydrophobic layers with and without the addition of different concentrations of E307 were recorded after 60 min and after 120 h of immersion of the electrode in a stagnant and naturally aerated simulated acid rain solution at pH 3. Figure 4 shows the polarisation curves of the different samples at shorter and longer time intervals of immersion of the samples in the selected corrosive medium. The electrochemical parameters obtained from the polarisation measurements, such as anodic Tafel slope (*β*_a_), cathodic Tafel slope (*β*_c_), corrosion current density (*i*_corr_), corrosion potential (*E*_corr_), and polarisation resistance (*R*_p_) and inhibition efficiency (*η*%), calculated according to Equations (1) and (2), are listed in Table 1 and Table 2.

(1) $$\eta \%=\left[1-\frac{i_{corr}^{\prime }}{i_{corr}}\right]\times 100$$

(2) $$\eta \%=\left[1-\frac{R_{p}}{R_{p}^{\prime }}\right]\times 100$$

where the designations *i*_corr_ and *R*_p_ were used for the measurements without inhibition effect, while the primed sizes $i_{\mathrm{corr}}^{\prime }$ and $R_{p}^{\prime }$ were used when the measurements were performed on the modified surfaces of SS type AISI 410S. The polarisation resistance was obtained from the linear polarisation within the potential range of ±20 mV with respect to *E*_corr_. Extrapolation of the Tafel line allowed the calculation of the corrosion current density *i*_corr_. 

In all potentiodynamic curves, whether the sample surface was treated or untreated, small current fluctuations were observed, which were more pronounced in the low current region. In any case, this may be related to the surface not yet stabilised, to diffusion processes of the electrolyte into the outer part of the passive layer on the untreated surface, or into a self-assembled hydrophobic layer. During the long-term immersion test (120 h), these oscillations were no longer present, which clearly proves the uniform surface of the metal samples. The bare surface of SS AISI 410S exhibits a relatively large corrosion current density of 5.371 μAcm^−2^ and corrosion potential of −0.504 V after 1 h of immersion, compared with the modified specimens with the self-assembled hydrophobic layer. The corrosion current density values were three orders of magnitude lower in the case of the (SA + 2.0% E307) layer, two orders of magnitude lower in the case of (SA + 1.0% E307), one order of magnitude lower in the case of (SA + 0.5% E307), and slightly lower in the case where the surface was modified only with a hydrophobic SA layer. 

After 120 h of immersion, the situation was somewhat different, as the passive layer formed on the stainless steel surface formed a barrier (protection) that reduced the corrosion current density of the base sample to 0.997 μAcm^−2^. The corrosion current density was also reduced significantly, from 0.0923 to 0.0025 μAcm^−2^, and from 0.243 to 0.008 μAcm^−2^ in the cases of (SA + 1.0% E307) and (SA + 0.5% E307), respectively. For the surface treated with SA only, the reduction was smaller, from 0.630 to 0.141 μAcm^−2^. In the case of (SA + 2.0%E307), there was practically no significant reduction in the corrosion current density (from 0.0025 to 0.0020 μAcm^−2^), which clearly indicates an extremely homogeneous structure of the fabricated layer. One could speculate that this is the layer with the least amount of micropores. 

The polarisation resistance increased significantly for the modified surfaces, reaching values typical of stable coatings (10^6^–10^9^ Ωcm^2^). A shift in the corrosion potential to more positive values (less negative) was also observed for all the modified surfaces. In the measurements after 1 h immersion in a corrosive medium, *E*_corr_ shifted up to 200 mV to the more noble values (SA + 2.0% E307), and up to 400 mV for the same composition of the hydrophobic layer in the case of a longer immersion time (120 h). The high corrosion resistance of the hydrophobic layers with the addition of E307 is also supported by the calculated values for inhibition efficiency, which were above 99%, even after 120 h of exposure in the selected corrosive medium.

#### 3.2.3. Electrochemical Impedance Spectroscopy (EIS)

Some general findings and observations are presented before starting with the detailed evaluation of the EIS measurements, which provide information on the most important properties of the as-prepared hydrophobic layers and their behaviour in the selected corrosive environment. 

All Nyquist diagrams showed a depressed capacitive loop over the entire frequency range. It was found that, after 1 h of immersion, semicircles were followed by a more or less straight line in the low frequency range, except in the case of the hydrophobic layer (SA + 2% E307). In measurements after 10, 25, and after 120 h, this phenomenon disappeared completely, indicating that the so-called water uptake had taken place and filled the pores with the electrolyte. Although the Nyquist diagrams appear to consist of a single depressed capacitive loop, which would dictate a single time constant, the Bode plots (phase angle versus log *f* frequency) clearly show multiple peaks in different frequency ranges (with multiple time constants in the EEC equivalent electrical circuit), which indicate different behaviours of the self-assembled hydrophobic layers and their resistance and stability. The low frequency response indicates the presence of diffusion pathways, which, at the same time, suggests the porosity of the as-prepared hydrophobic layers. This phenomenon varied from case to case, i.e., at different concentrations of the added E307. All the selected systems were in common in that both the charge transfer resistance *R*_ct_ and the total polarisation resistance *R*_p_ increased with the increasing immersion time. This indicates that either the stability of the as-prepared hydrophobic layers or, in the case of pores, that the penetration of the electrolyte into the pore during the exposure time did not come into contact with the underlying metal matrix, since the EDAX measurements did not indicate the presence of oxygen, which would confirm the formation of a passive layer on the SS AISI 410S. However, the parameters for the resistivity of the film and the pores *R*_f_ and *R*_p_, as well as their capacitance values, also changed during the exposure period.

**Figure 5:**
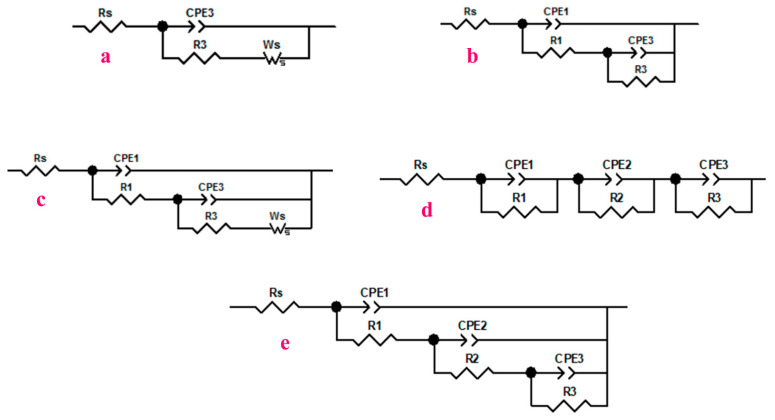
Equivalent circuits for EIS results for the bare and as-prepared hydrophobic surfaces of SS AISI 410S samples. (**a**) Randles EEC, bare surface SS AISI 410S after 1 h and 10 h of immersion (**b**) bare surface SS AISI 410S after 25 h and120 h of immersion and as-prepared hydrophobic surfaces (SA) after 10 h, 25 h and 120 h of immersion (**c**) as-prepared hydrophobic surfaces (SA) after 1 h of immersion (**d**) as-prepared hydrophobic surfaces (SA + 0.5% E307) and (SA + 1.0% E307) after 1 h–120 h of immersion and also (SA + 2.0% E307) after 120 h of immersion (**e**) as-prepared hydrophobic surfaces (SA + 2.0% E307) after 1h-25h of immersion.

Fitting of the experimental impedance spectra was performed using appropriate electrical equivalent circuits (EEC). Figure 5 shows the EECs used, which yielded the fitting curves that best matched the experimental values. As is well known, the elements of the EEC consist of a combination of resistors (*R*) and the constant phase element (CPE) in series with *R*_s_ (the resistance of the electrolyte). The experimental data showed a frequency dispersion; thus, a modelling element with frequency dispersion behaviour, a constant phase element (CPE), was used, i.e., the shape of the depressed semicircles indicated a non-ideal capacitive behaviour. For this reason, a constant phase element (CPE) must be used in the modelling to achieve an optimal fit. The heterogeneity of the surface, resulting from the surface roughness, porosity or inhomogeneous distribution of the surface properties of the electrode, is the main reason for the phenomenon of frequency depression, which was then taken into account in the CPE.

The impedance of the CPE is expressed as $Z_{CPE}=Q^{-1}{\left(j\omega \right)}^{-n}$ [57,58], where ***j*** is an imaginary unit, ***ω*** is an angular frequency, *Q* is a proportionality coefficient, and *n* is an exponent related to the phase shift. When *n* = 1, *Q* represents the pure capacitance, while for *n* ≠ 1, it can be a sign of surface heterogeneity or further charge transfer reactions [59]. The value for the parameter *Q* of the *Z*_CPE_ varied, mostly regarding the concentration. The capacitances in μFcm^−2^ were calculated using the following relationship Cdl=(Q·R1−n)1n [29,58]. The basics of the EIS measurements are not discussed in detail here, as they can be found in any book or articles describing this non-destructive and very useful measurement technique, which provides a great deal of information about the condition and behaviour of a surface relative to its surroundings [57,58,60,61,62].

##### Bare Surface

**Figure 6:**
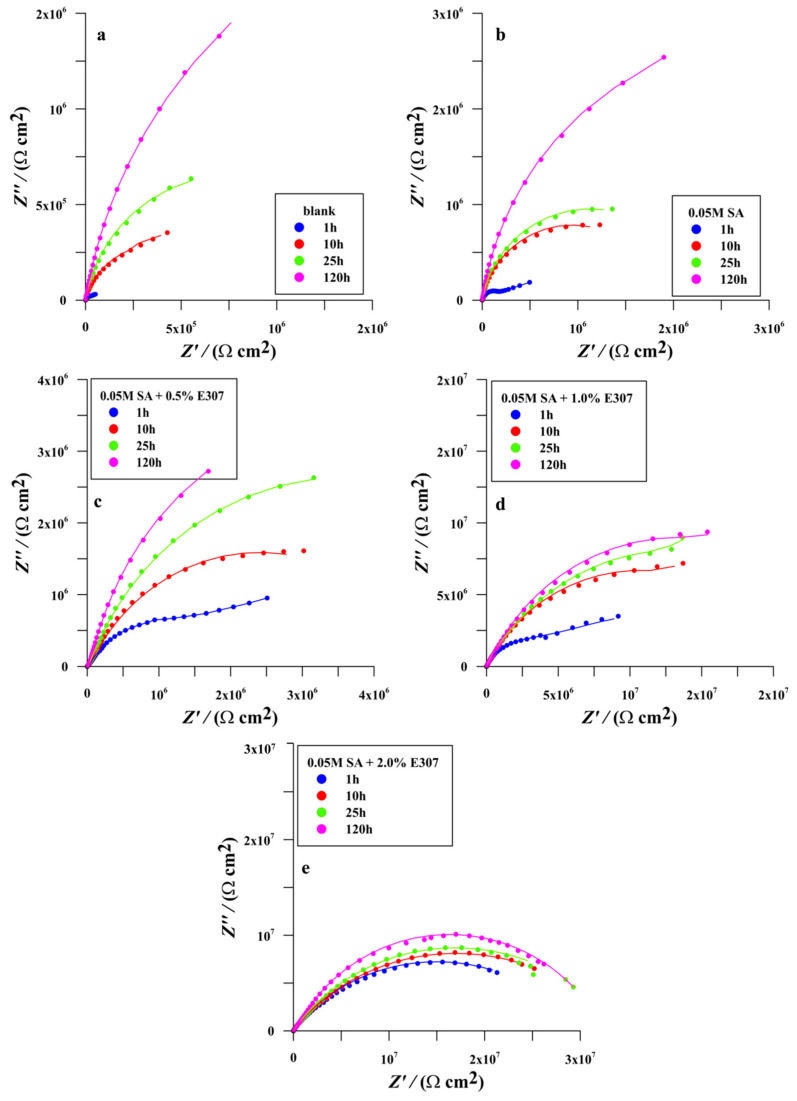
EIS Nyquist plots of SS AISI 410S samples for bare and modified surfaces measured after 1, 10, 25, and after 120 h of immersion at their open-circuit potential in a simulated solution of acid rain with pH = 3 at 25 °C: (**a**) Blank, (**b**) SA, (**c**) (SA + 0.5% E307), (**d**) (SA + 1.0% E307) and (**e**) (SA + 2.0% E307) (measured data-dotted symbols and fitted data-solid lines).

**Figure 7:**
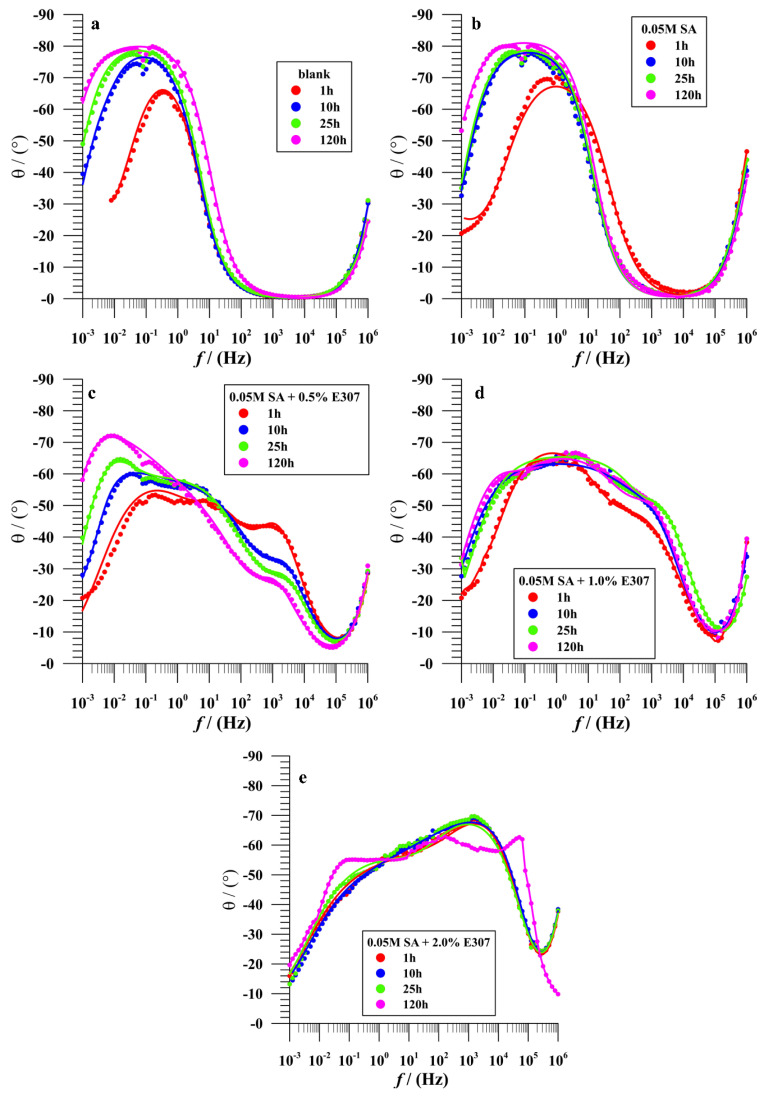
Bode-phase angle plots of SS AISI 410S samples for bare and modified surfaces measured after 1, 10, 25, and after 120 h of immersion at their open-circuit potential in a simulated solution of acid rain with pH = 3 at 25 °C: (**a**) Blank, (**b**) SA, (**c**) (SA + 0.5% E307), (**d**) (SA + 1.0% E307) and (**e**) (SA + 2.0% E307) (measured data-dotted symbols and fitted data-solid lines).

**Figure 8:**
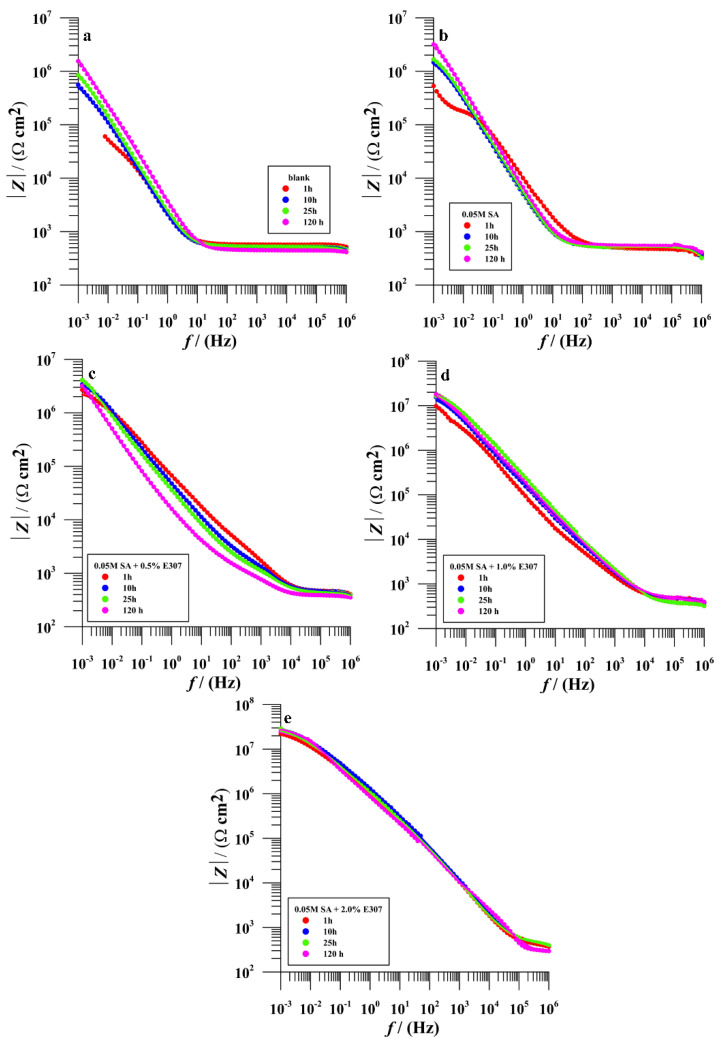
Bode-|Z|-module plots of SS AISI 410S samples for bare and modified surfaces measured after 1, 10, 25, and after 120 h of immersion at their open-circuit potential in a simulated solution of acid rain with pH = 3 at 25 °C: (**a**) Blank, (**b**) SA, (**c**) (SA + 0.5% E307), (**d**) (SA + 1.0% E307) and (**e**) (SA + 2.0% E307) (measured data-dotted symbols and fitted data-solid lines).

From the Nyquist diagram (Figure 6a), it can be seen that the typical semicircular capacitive loops increased significantly with increasing immersion time, especially after 10 h, which was due to the formation of a passive layer, a typical phenomenon for stainless steels. This fact was also confirmed by the two Bode diagrams (Figure 7a and Figure 8a). The plateau broadened with immersion time from 100 to 0.01 Hz, with the negative phase increasing from an initial value of −60° to −80° and the *n*-parameter (CPE) approaching 0.9 after 10 h of immersion, indicating a predominantly capacitive behaviour (Figure 7a). Similarly, the plateau of the maximum negative phase value lay between 1.0 and 0.1 Hz at 1 h immersion, and extended from 1.0 to 0.05 Hz at 120 h of immersion. Moreover, the slope of log *|Z|* versus log *f* was close to −1 (Figure 8a), indicating strong barrier properties of the forming surface oxide. In the case of the surface SS without a formed hydrophobic coating, a modified Randles electrical equivalent circuit with a Warburg element (Figure 5a) was used for measurements after 1 and 10 h immersion in the corrosive medium. The Nyquist diagram (Figure 6a) shows a straight line in the low-frequency region typical of the Warburg impedance associated with diffusion processes, which can be justified by the fact that the passive layer was still incomplete and allowed the electrolyte to penetrate. The difference in Warburg values (0.019 S cm^−2^ s^1/2^ after 1 h and 0.141 mS cm^−2^ s^1/2^ after 10 h of immersion) also clearly shows that the passive layer was building up and reducing the possibility of electrolyte penetration. However, after the Warburg element was included in the EES, the agreement of the fitting curve with the experimental points improved significantly, as shown by the value of chi-square (χ^2^), which has values between 0.0003 and 0.0006. As we proceeded, the impedance curves did not differ significantly for the next two immersion times. It could be said that we had only one time constant, but χ^2^ increased by an order of magnitude when we used an electrical equivalent circuit according to Randles [63,64]. For this reason, an EEC with two RC time constants was included in the fitting process after 25 and 120 h of immersion (Figure 5b).

In these two EECs (Figure 5a,b), *R*_s_ refers to the resistance of the electrolyte solution, *R*_1_ represents the resistance of the film (passive film), and CPE_1_ is the constant phase element associated with the nonporous regions of the film. The electrical element *R*_3_ represents the charge transfer resistance associated with the corrosion process, and CPE_3_ is the constant phase element (CPE) associated with the electrochemical double layer at the metal/electrolyte interface and *W*_s_ represents Warburg element (*W*_s_−short “semi-infinite” diffusion). The results show an increase in the charge transfer resistance or polarisation resistance *R*_p_ with increasing immersion time, by two orders of magnitude between 1 and 120 h immersion (long-term test), from 62.2 kΩcm^−2^ to 5.09 MΩcm^−2^, while the capacitance decreased by an order of magnitude from 144.2 to 98.0 144.2 μFcm^2^, also clearly confirming the formation of a passive layer or surface oxide, which has been addressed by many authors in their work [65,66].

##### Surface Modified in Stearic Acid (SA)

In the case where the surface was modified with a self-assembled hydrophobic layer formed by immersion in an ethanolic solution of SA, the impedance curves for all immersion times did not differ significantly and were similar to the curves obtained for the measurements on the unmodified surface explained in the previous paragraph. In this particular case, the Nyquist diagram (Figure 6b) showed the presence of diffusion or penetration of electrolytes only when measured after 1 h of immersion in the selected corrosive medium, but the value of the Warburg element was three orders of magnitude lower (0.068 mS cm^−2^ s^1/2^) than for the unmodified surface of SS under the same immersion conditions (after 1 h). In this case, the EEC shown in Figure 5c was used. For the other longer immersion times, where the diffusion tail was absent from the Nyquist diagrams, indicating improved surface protection by the hydrophobic layer, the EEC with two time constants and without the Warburg element was used (Figure 5b). The onset of capacitive behaviour began at the higher frequency of 10^3^ Hz. The region with the maximum phase angle value (−80°) during the long-term test, after 120 h of immersion, also extended from nearly 10.0 Hz to the millihertz range (Figure 7b). The charge transfer resistance increased and was slightly higher than for the unmodified SS surface, on which the passive layer (metal oxide) was supposed to form. The capacitance of the double layer was an order of magnitude lower (Table 2) than that of the unmodified surface, indicating that the hydrophobic layer was an effective barrier to the penetration of the electrolyte into the metal matrix itself, which also confirmed the increase in polarisation resistance with prolonged exposure. 

##### Surface Modified in Surface Modified in the Mixtures of (SA + x%E307, x = 0.5%, 1.0% and 2.0%) 

When E307 was added to the ethanolic solution of SA, during the formation of a hydrophobic layer, there was a significant change in the response in the impedance diagrams, especially in the Bode diagram, which shows the change in phase angle as a function of the logarithm of frequency (Figure 7c–e). The capacitive response started already in the high frequency range of 10^5^ Hz, and one could say that such a response extended up to 0.001 Hz, i.e., from the high to the low frequency range. The so-called “three peaks” were visible, especially when the hydrophobic layer was formed by immersion in the solutions (SA + 0.5% E307) and (SA + 1.0% E307), and for the (SA + 2.0% E307) after 120 h of immersion in the chosen corrosive medium. Such a surface response requires three time- constants in the EEC to fit the model to the experimental values. In the case of (SA + 0.5% E307), the fit was better, even if four time constants were used in the EEC. This could be due to the less homogeneous structure of the resulting layer, possible separation or delamination of individual layers, but this is all a possible assumption. For this reason, we treated all three systems with three time constants, but with two different types of EEC. In the case of (SA + 0.5% E307) and (SA + 2.0% E307) after 120 h of immersion, we used EEC R(QR)(QR)(QR), (Q = CPE) (Figure 5d).

The EEC used to analyse the EIS data of the modified surfaces in (SA + 1.0% E307) and (SA +2.0% E307) after 1, 10, and 25 h is written schematically as R(Q(R(Q(R(QR))) and is shown in Figure 5e. In the EEC used to fit the experimental data (Figure 5d,e), *R*_s_ represents the resistance of the solution, *R*_1_ represents the resistance of the prepared hydrophobic layer (superhydrophobic layer), and *R*_2_ represents the resistance of the pores due to the possible penetration of electrolytes. As soon as organic self-assembled coatings/layers are involved, the porosity of the self-assembled protective layer and the presence of smaller or larger defects in the layer can hardly be avoided, which means that there are places where the solution takes place, the speed of which depends on the geometrical parameters of the pores themselves, and *R*_3_ represents the charge transfer resistance. For each of the above resistances, the corresponding capacitance, or CPE, must be specified after the EEC (Figure 5c,d) (CPE_1_-hydrophobic layer, CPE_2_-pore and CPE_3_-double layer).

As mentioned earlier, the Bode diagrams showing the change in phase angle as a function of log frequency clearly show the existence of three time constants in the case of the modified SS AISI 410S surface (Figure 7c–e). The time constant in the high frequency range from 10^6^ to 10^3^ Hz represents the properties of the hydrophobic protective layer, and its values were highest for the hydrophobic layer (SA + 2.0% E307) near −75° (Figure 7e). These values were lower for (SA + 1.0% E307) and (SA + 0.5% E307). From Figure 7d, which shows the change in phase angle for (SA + 1.0% E307), it can be seen that the highest value was reached after 25 and 120 h and was close to −70°. This phenomenon could be attributed to the possible higher porosity of the formed layer, i.e., lower resistance values of the hydrophobic film could indicate that the electrolyte had penetrated into the pores. Due to their own configuration, perhaps cone-shaped, they did not allow the penetration of the electrolyte into the metal matrix, which was confirmed by the high values of the pore resistance (Table 2).

In the case of (SA + 0.5% E307), it can be seen that the phase angle increased from the higher frequency ranges to the lowest frequencies (Figure 7c). The maximum value of the phase angle was in the range from 0.1 to 0.001 Hz, which could be due to diffusion processes enabled by the porosity of the hydrophobic layer, quite similar to the previous case. Considering the fact that also in this composition the total polarisation resistance increased, it is possible to speculate again about the presence of a suitable pore configuration (cone shape), which does not allow for or slows down the access of electrolytes to the metal matrix strongly. The mid-frequency range from 10^3^ to 0.1 Hz shows the response of the pores in the hydrophobic layer. The resistance values for all modified surfaces showed an increasing trend. Comparing the corrosion resistance of the formed hydrophobic layer for different additions of E307, first, after 1 h exposure, it can be based on the comparison of the polarisation resistance *R*_p_ (the sum of all three *R*_p_ = *R*_1_ + *R*_2_ + *R*_3_) [67]. It is clear that the *R*_p_ values at (SA + 2.0% E307) was one order of magnitude higher, obviously indicating the quality of the self-assembled hydrophobic layer on the surface of the AISI 410S SS in the acidic corrosive medium, namely, *R*_p_ (SA + 2.0% E307) = 26.2 MΩcm^2^, followed by *R*_p_ (SA + 1.0% E307) = 5.8 MΩcm^2^ and *R*_p_ (SA + 0.5% E307) = 2.9 MΩcm^2^. The values of film and pore resistance (*R*_1_ and *R*_2_) and their capacitance also showed similar behaviour (resistances increased, while capacitances decreased). The highest resistance and the lowest capacitance values confirmed the high inhibition properties of the as-prepared hydrophobic film in the initial state itself. In the long-term test, the values of *R*_2_, *R*_3_ and *R*_p_ showed an increasing trend for all the selected systems. Some changes and fluctuations were observed in the values of film resistance *R*_1_. For the as-prepared hydrophobic layer in the initial state (SA + 0.5% E307), the film resistance decreased with time, but without dramatic effects on the trend of the polarisation resistance *R*_p_. It increased and showed values very similar to those when the surface was modified with SA only. This could indicate that this E307 concentration was too low, or that the layer was not compact enough to achieve higher *R*_p_ values (as in the case of 1.0% or 2.0% added E307). In the case of (SA +1.0% E307), *R*_1_ values were an order of magnitude higher, but during the long-term test, these values fluctuated (Table 2). It is quite unusual that the *R*_2_ values (pore resistance) are highest for this composition during the long-term test. This could be due to the fact that the pores were filled with electrolyte and there was no direct path to the underlying metal matrix. At the highest added E307 concentration (SA + 2.0% E307), *R*_1_ was another order of magnitude higher, while *R*_2_ had a lower value compared to (SA + 1.0% E307). This could be due to the lower presence of pores, which was also reflected in the highest *R*_p_ values throughout the immersion test from 1 to 120 h. The impedance modulus |Z| (Figure 8a–e) at low frequencies for the bare and modified samples of AISI 410S SS showed an increasing trend for all the selected systems, including the bare surface. In the case of (SA + 2.0% E307), it looked as if the curves almost copied each other, as they looked invariant, i.e., constantly stable at all immersion times (Figure 8e). In addition, it can be observed how the capacitive area increased with the addition of E307 towards the modified surface. For the bare surface and the surface modified only with SA, the capacitive range started in the middle frequency range of 10 and 100 Hz, respectively, while, for the surfaces modified with the as-prepared hydrophobic layer with the composition (SA + x% E307), it was already between 10^4^ and 10^5^ Hz, indicating a higher quality level of the protective hydrophobic layer. The capacitance values were in agreement with those obtained for the resistance. It was expected that the capacitance of film *C*_1_ would be lowest in the case of the layer (SA + 2.0% E307), ranging from 0.025 μFcm^−2^ after 1 h of exposure to 0.0065 μFcm^−2^ after 120 h of exposure, and highest in the case of the bare surface, 62.67 μFcm^−2^. The capacitance of the pores *C*_2_ increased with immersion time, indicating water uptake. However, these values were lowest again in the case of the layer (SA + 2.0% E307) with μFcm^−2^ (after 1 h) and up to 0.12 μFcm^−2^. The same trend of lowest and decreasing values was seen in the case of the capacitance of double layer *C*_3_, which decreased from 1.24 μFcm^−2^ (after 1 h) to 0.214 μFcm^−2^ (after 120 h). All other values are listed in Table 2. Although the calculated values of the inhibition efficiency were high for all three modified surfaces with added E307, based on the impedance parameters obtained, it was clearly observed that the as-prepared self-assembled hydrophobic layer (SA + 2.0% E307) had the highest quality and was the most stable compared to the other two. All three were stable after 120 h immersion in the acidic corrosive media with a pH value of 3 (simulated acid rain). The layer (SA + 2.0% E307) can be predicted to last longer than 120 h. 

### 3.3. Surface Characterisation/Characterisation of Coating Material

#### 3.3.1. ATR–FTIR Analysis

**Figure 9:**
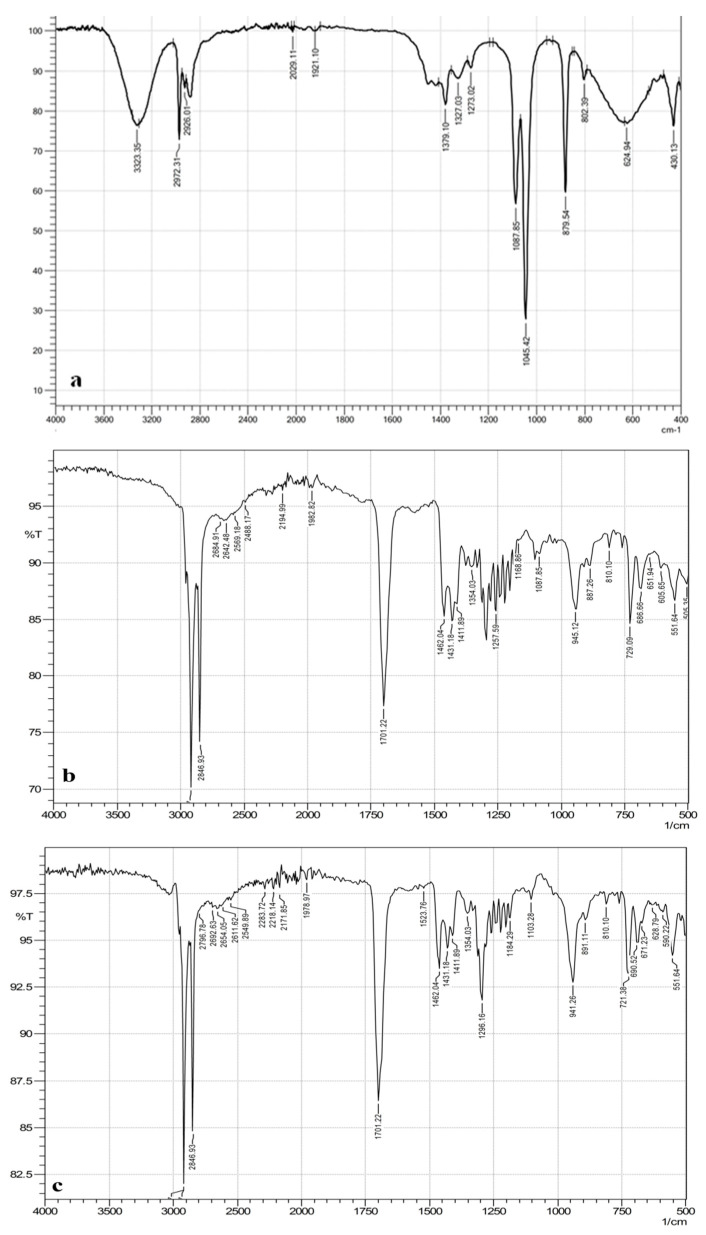
ATR-FTIR spectra (**a**) for pure E307, (**b**) modified surface of SS type AISI 410S in ethanolic solution of SA with the addition of 2.0% of E307 for one hour [27], (**c**) modified surface of SS type AISI 410S in ethanolic solution of SA with the addition of 2.0% of E307 after 120 h of immersion in simulated solution of acid rain with pH = 3 (transmittance vs. wavenumber).

The chemical composition and binding states of the prepared hydrophobic layers were investigated in our previous study FTIR-ATR, in which we also confirmed the presence of alpha-tocopherol (E307) in the modified surfaces AISI 410S SS [27]. Several authors have confirmed the characteristic absorption bands of pure alpha-tocopherol at the following wavenumbers (Table 3) [68,69,70,71,72,73,74]:

Considering that a mixture of acid and alcohol was used to form the hydrophobic protective layers, the appearance of the absorption band for the ester functional group at 1710 cm^−1^ was to be expected, while the −OH stretching vibrations (3300–3100 cm^−1^) were no longer present. Figure 9a shows the ATR-FTIR spectrum of the alcoholic mixture (SA + 2.0% E307), while Figure 9b,c show the ATR-FTIR spectra of the as-prepared hydrophobic layer on the SS samples after 1 h immersion in an ethanolic solution of (SA +2.0% E307) and after 120 h immersion in a simulated solution of acid rain with pH = 3, respectively. The purpose of the last two spectra was to show the difference or similarity in the structure of the as-prepared hydrophobic layer on the samples SS after 1 h immersion in an ethanolic solution of (SA + 2.0% E307) and after 120 h immersion in a simulated solution of acid rain with pH = 3. The high values of inhibition efficiency, even with prolonged exposure to the corrosive medium, were certainly in agreement with Figure 9c, which was not significantly different from an ATR- FTIR spectrum of an as-prepared hydrophobic layer on SS immediately after formation (Figure 9b), indicating the high stability of this layer in the chosen corrosive medium. The difference was in the transmittance value, which was higher, and which could be attributed to possible thinning of the layer.

#### 3.3.2. SEM- EDAX

The EDAX analysis can be used to determine the elemental composition of individual points, or to map out the lateral distribution of elements from the imaged area, and its application has been extended to study organic-based specimens or organic layers on certain substrates [75,76]. We were interested in a possible change in the carbon content of the modified surface during prolonged exposure to a corrosive environment, which could also provide additional information about the state of the self-assembled hydrophobic layer or indicate specific changes on the surface. For this reason, we performed an EDAX analysis of the untreated sample (without the layer), immediately after the as-prepared self-assembled layer was formed, and after 120 h of immersion of the SS sample in the corrosion medium. The EDAX analysis clearly shows a significant increase in the carbon content on the surface of the SS AISI 410S after the formation of the prepared hydrophobic layer, which was increased further by the addition of E307, while, in contrast, on the bare surface of the stainless steel, the presence of carbon was negligible. The results of the EDAX analysis showed a slightly lower carbon content, which could indicate some changes in the hydrophobic layer itself (Table 4). Considering the high inhibition efficiency achieved even after 120 h immersion, it could be suggested that this decrease was not yet dramatic.

## 4. Conclusions

The electrochemical measurements revealed a high level of barrier properties provided by the as-prepared self-assembled superhydrophobic layers with flower-like structures (cornflower) for the period of contact with the aggressive medium (urban acid rain pH = 3) from 1 to 120 h of exposure. The EIS and polarisation studies showed that the corrosion resistance (polarisation resistance) of the superhydrophobic surface was in the range of 10^6^–10^7^ Ωcm^2^, which corresponds to the typical values of stable coatings (10^6^–10^9^ Ωcm^2^) and increased with increasing the immersion time. In addition, the morphological (SEM) and chemical composition analyses (FTIR) showed that the superhydrophobic surfaces were stable to corrosive media, as the morphological features, as well as the molecular structures, did not change significantly after long-term corrosion tests. Based on our extended research, we can conclude that the as-prepared self-assembled superhydrophobic layers consisting of (SA + x% E307, x > 0.5) present potential for use as corrosion protection, both in the 3% NaCl and in the acidic media with pH ≥ 3. In the future, it would be necessary to focus on the effects of UV radiation on the resistance of the as-prepared self-assembled superhydrophobic layers; in addition, resistance to mechanical damage, durability and other impacts would have to be carried out, in particular, in accordance with the relevant standards.

## Figures and Tables

**Table 1:** Kinetic parameters for corrosion of SS type AISI 410S obtained from potentiodynamic polarisation curves for the bare and modified surfaces in simulated solution of acid rain with pH = 3 at 25 °C after 1 h of immersion and after 120 h testing.

Corrosive Media Acid Rain, pH = 3		*i*_corr_ (μAcm^−2^)	*E*_corr_ (V/SCE)	*R*_p_ (MΩ cm^2^)	*β*_c_ (mV/dec)	*β*_a_ (mV/dec)	*% η_i_*_corr_	*% η_R_*_p_
after 1 h bare surface AISI 410S								
		5.731	−0.504	0.020	−120.83	141.02		
modified surface	wt% E307							
	0*	0.630	−0.448	0.120	−127.3	321.28	85.8	83.4
	0.5	0.244	−0.395	0.271	−143.9	313.31	95.5	92.7
	1.0	0.092	−0.387	0.528	−149.6	332.21	98.3	96.2
	2.0	0.0025	−0.275	9.010	−146.0	339.37	99.9	99.8
after 120 h, bare surface AISI 410S								
		0.997	−0.402	0.054	−121.3	272.5		
modified surface	wt% E307							
	0*	0.141	−0.193	0.317	−142.2	232.7	85.8	83.1
	0.5	0.008	−0.141	3.249	−141.2	292.1	99.2	98.3
	1.0	0.0025	−0.123	11.730	−137.7	307.2	99.7	99.5
	2.0	0.0020	−0.001	18.690	−157.3	315.4	99.8	99.7

0* modified surface AISI 410S within 0.05 M SA, 0.05 M SA + x wt% E307.

**Table 2:** Electrochemical impedance parameters (EIS fitting results) for SS AISI 410S (for the bare and modified surfaces) after immersion in simulated solution of acid rain with pH = 3 for different times at 25 °C.

Corrosive Media Acid Rain, pH = 3		*R*_s_ (Ωcm^2^)	*R*_1_ (kΩcm^2^)	*n*_1_	*C*_1_ (µFcm^−2^)	*R*_2_ (MΩcm^2^)	*n*_2_	*C*_2_ (µFcm^−2^)	*R*_3_ (MΩ cm^2^)	*n*_3_	*C*_3_ (µFcm^−2^)	*R*_p-EIS_ (MΩ cm^2^)	*% η_R_*_p-EIS_
bare surface	1 h	165.5	/	/	/	/	/	/	0.048	0.862	124.30	0.120	
	10 h	163.2	/	/	/	/	/	/	0.646	0.850	259.21	0.271	
	25 h	159.7	0.272	0.892	62.67	/	/	/	0.801	0.392	17.29	0.528	
	120 h	155.5	0.387	0.901	60.15	/	/	/	1.264	0.406	1.22	9.010	
modified surface SA	1 h	241.5	0.540	0.956	3.14	/	/	/	0.219	0.781	26.91	0.220	77.91
	10 h	236.1	0.791	0.903	15.08	/	/	/	1.898	0.829	41.06	1.899	97.44
	25 h	232.8	0.657	0.908	13.70	/	/	/	2.257	0.895	22.23	2.258	97.85
	120 h	240.0	0.617	0.925	11.14	/	/	/	6.047	0.911	16.44	6.048	99.20
modified surface (SA + 0.5%E307)	1 h	339.8	1.954	0.782	0.220	0.021	0.663	3.044	2.922	0.761	8.20	2.945	98.35
	10 h	335.1	0.826	0.766	0.209	0.064	0.592	12.24	4.738	0.757	21.45	4.803	98.99
	25 h	349.5	0.603	0.760	0.208	0.132	0.602	20.30	6.311	0.853	31.06	6.444	99.25
	120 h	339.0	0.350	0.838	0.601	0.158	0.523	19.12	8.280	0.906	47.01	8.437	99.42
modified surface (SA + 1.0%E307)	1 h	346.2	9.893	0.731	0.266	0.005	0.731	0.266	5.830	0.803	1.865	5.845	99.17
	10 h	340.4	15.102	0.726	0.219	3.850	0.768	0.625	16.212	0.768	0.625	20.081	99.76
	25 h	341.6	8.492	0.755	0.109	7.900	0.744	0.660	16.432	0.615	0.622	24.322	99.80
	120 h	350.2	10.724	0.761	0.150	6.901	0.722	1.249	20.105	0.890	0.746	27.010	99.82
modified surface (SA + 2.0%E307)	1 h	475.3	101.1	0.808	0.025	0.326	0.651	0.052	25.801	0.538	1.240	26.231	99.81
	10 h	484.8	220.6	0.789	0.034	1.094	0.658	0.080	26.180	0.694	0.401	27.492	99.82
	25 h	473.9	225.4	0.777	0.044	3.474	0.594	0.080	28.121	0.699	0.349	31.820	99.85
	120 h	479.5	563.9	0.990	0.007	4.074	0.668	0.120	28.592	0.713	0.214	33.231	99.86

**Table 3:** ATR-FTIR bands assignment of alpha-tocopherol (E307) [68,69,70,71,72,73,74].

3473 cm^−1^	−OH stretching band
2927 cm^−1^ 2868 cm^−1^	symmetric and asymmetric stretching vibrations of CH_2_ and CH_3_
1461 cm^−1^	asymmetric bending of the phenyl skeleton and methyl group
1378 cm^−1^	symmetric bending of the methyl group
1262 cm^−1^	−CH_2_
1086 cm^−1^	plane bending of phenyl group
919 cm^−1^	for trans = CH_2_ stretching
1207 cm^−1^ 1110–1107 cm^−1^ 1068–1062 cm^−1^	stretching vibration of C−O

**Table 4:** EDAX analysis of the bare surface and modified surfaces of SS type AISI 410S after one-hour immersion in an ethanolic solution of SA [27] and (SA + 2.0% of E307) [27] and after 120 h of immersion in a simulated solution of acid rain with pH = 3.

		After Fabrication				After 120 h		of Immersion	
Bare Surface		Modified Surface (SA)		Modified Surface (SA + 2%E307)		Modified Surface (SA)		Modified Surface (SA + 2%E307)	
**Element**	**wt%**	**Element**	**wt%**	**Element**	**wt%**	**Element**	**wt%**	**Element**	**wt%**
C K	0.06	C K	43.04	C K	51.02	C K	41.74	C K	47.88
Si K	0.55	Si K	0.37	Si K	0.17	Si K	0.41	Si K	0.25
Cr K	15.74	Cr K	7.52	Cr K	6.71	Cr K	8.02	Cr K	7.37
Fe K	83.11	Fe K	49.08	Fe K	42.11	Fe K	49.83	Fe K	44.51
Total	100.00	Total	100.00	Total	100.00	Total	100.00	Total	100.00

## Data Availability

The data presented in this study are available on request from the corresponding author.
